# Vasoactive Inotropic Score as a Prognostic Factor during (Cardio-) Respiratory ECMO

**DOI:** 10.3390/jcm11092390

**Published:** 2022-04-24

**Authors:** Stany Sandrio, Joerg Krebs, Eva Leonardy, Manfred Thiel, Jochen J. Schoettler

**Affiliations:** Department of Anesthesiology and Critical Care Medicine, University Medical Centre Mannheim, Medical Faculty Mannheim, University of Heidelberg, Theodor-Kutzer-Ufer 1-3, 68165 Mannheim, Germany; joerg.krebs@umm.de (J.K.); e.leonardy@gmx.de (E.L.); manfred.thiel@medma.uni-heidelberg.de (M.T.); jochen.schoettler@umm.de (J.J.S.)

**Keywords:** vasoactive inotropic score, extracorporeal membrane oxygenation (ECMO), V-V ECMO, V-VA ECMO, cannulation strategy

## Abstract

The vasoactive inotropic score (VIS) is calculated as a weighted sum of all administered vasopressor and inotropic medications and quantifies the amount of pharmacological cardiovascular support in patients with the most severe combined cardiopulmonary failure supported with extracorporeal membrane oxygenation (ECMO). This study evaluated (1) whether VIS prior to the initiation of ECMO is an independent predictor of survival in these patients and (2) whether VIS might guide the selection of the appropriate extracorporeal cannulation modality (Veno-Venous ‘V-V’ or Veno-VenoArterial ‘V-VA’). In this study, 39 V-VA and 182 V-V ECMO runs were retrospectively analyzed. VIS immediately prior to ECMO initiation (pre-ECMO) was 40 (10/113) in all patients, 30 (10/80) in patients with V-V ECMO and 207 (60/328) in patients with V-VA ECMO. Pre-ECMO VIS was an independent predictor of survival in univariate (AUC = 0.68, *p* = 0.001) and multi-variable analyses (*p* = 0.02). Pre-ECMO VIS was clearly associated with mortality (*p* = 0.001) in V-V ECMO group; however, V-VA ECMO disrupted this association (*p* = 0.18). Therefore, in conjunction with echocardiography, VIS might assist in selecting the appropriate ECMO cannulation strategy as patients with a pre-ECMO VIS ≥ 61.4 had significantly lower odds of survival compared to those with lower VIS.

## 1. Introduction

In the most severe cases of acute respiratory distress syndrome (ARDS) with refractory hypoxemia [1], Veno-Venous (V-V) extracorporeal membrane oxygenation (ECMO) has been proposed as an alternative support to maintain oxygenation [2,3,4]. However, respiratory insufficiency can also be associated with considerable hemodynamic compromise that requires vasopressor and inotropic support [5]. In these circumstances, the optimal extracorporeal modality has yet to be defined [6].

V-V ECMO might improve the right heart function through a reduction in pulmonary vascular resistance but does not provide circulatory support during left or right ventricular failure or during severe vasoplegia. In these cases, Veno-Arterial (V-A) and Veno-VenoArterial (V-VA) ECMO have been favored [6,7]. V-VA ECMO is typically applied in patients on V-A ECMO for primary cardiogenic shock who develop secondary respiratory failure due to pulmonary congestion, leading to an upper body hypoxia [8]. This, however, should be clearly separated from an approach which utilized V-VA ECMO for primary respiratory failure and secondary acute cor pulmonale or septic cardiomyopathy [9].

Since there are no objective criteria to select the appropriate ECMO modality (V-V or V-VA) on patients with primary respiratory and concomitant cardio-circulatory failure, clinicians usually base the cannulation strategy on personal preferences. Patients without cardio-circulatory failure are managed with V-V ECMO and patients with significant hemodynamic impairment are supported with V-A or V-VA ECMO.

The vasoactive inotropic score (VIS) provides a descriptive tool to quantify pharmacological cardio-circulatory support. VIS is calculated as a weighted sum of all administered vasoactive inotropic agents [10]. Initially, VIS assessed only dopamine, dobutamine, epinephrine, norepinephrine, milrinone and vasopressin [10]. Due to frequent application of levosimendan, it is increasingly integrated in VIS calculation [11].

VIS has been validated in both pediatric and adult cardiac surgery [10,12]. In both reports, the maximum VIS within the first 24 h after cardiac surgery is an independent predictor of post-operative morbidity and mortality [10,12]. The European Society of Pediatric and Neonatal Intensive Care incorporates a VIS greater than 200 points, lactate level greater than 8 mmol/L and myocardial dysfunction in their bedside septic shock scoring system [13]. Although their VIS calculation did not integrate levosimendan, a VIS greater than 200 is associated with mortality and the requirement of extracorporeal life support [13]. Furthermore, Surviving Sepsis Campaign International Guidelines suggest V-A ECMO as a rescue therapy in children with refractory septic shock [14].

In this study, we aimed (1) to evaluate whether VIS immediately prior to ECMO initiation (pre-ECMO VIS) is an independent predictor of survival, (2) to evaluate whether pre-ECMO VIS is an effective determinant to select appropriate extracorporeal modality (V-V or V-VA ECMO) and (3) to assess the impact of V-VA ECMO on mortality in patients with high pre-ECMO VIS.

## 2. Materials and Methods

### 2.1. Data Acquisition, Inclusion and Exclusion Criteria

This retrospective study was approved by the institutional ethics committee (Medizinische Ethikkomission II, University Medical Centre Mannheim, Medical Faculty Mannheim of the University of Heidelberg, registration number 2019-834R) and registered in the German Clinical Trials Register (DRKS00027491).

**Figure A1:**
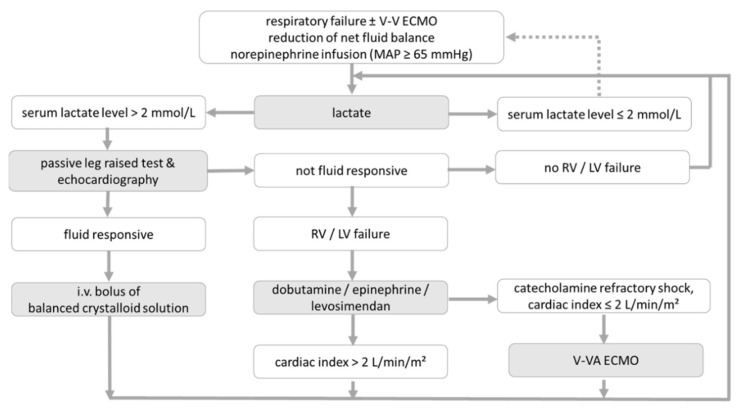
Clinical workflow on patients with respiratory failure. ECMO: Extracorporeal Membrane Oxygenation; V-V: Veno-Venous; V-VA: Veno-VenoArterial; MAP: mean arterial pressure; RV: right ventricular; LV: left ventricular; i.v.: intravenous.

The study was completed in the intensive care unit of the Department of Anesthesiology and Critical Care Medicine, University Medical Centre Mannheim, Germany. A review of medical records was performed to identify patients who had been supported with V-V and V-VA ECMO between November 2014 and July 2020. Our institutional management strategy for patients on ECMO support due to primary respiratory failure is outlined in the Appendix A and Figure A1.

We included all patients who received V-V or V-VA ECMO due to severe (cardio-) pulmonary failure irrespective of their underlying cause or disease. Patients who required ECMO support for reasons other than (cardio-) pulmonary failure (e.g., ECMO as intraprocedural support during elective aortic repair, extracorporeal life support during resuscitation) were excluded from this study.

The Simplified Acute Physiology Score II (SAPS II) and the Sequential Organ Failure Assessment (SOFA) score were calculated within the first 24 h of ICU stay [15,16]. VIS was assessed at ICU admission, immediately prior to ECMO initiation (pre-ECMO), during the first two days of ECMO and at the termination of ECMO support (ECMO end). VIS was calculated as:

VIS = dopamine (µg/kg/min) + dobutamine (µg/kg/min) + 100 × epinephrine (µg/kg/min) + 100 × norepinephrine (µg/kg/min) + 10 × milrinone (µg/kg/min) + 10,000 × vasopressin (units/kg/min) + 50 × levosimendan (µg/kg/min) [11].

### 2.2. Statistical Analysis

The primary outcome was ICU mortality. Categorical variables were reported as frequency of observation (*n*, %) and analyzed using Pearson’s chi-squared test. Continuous variables were reported as medians (interquartile range Q1–Q3) and evaluated with the Mann–Whitney U-Test method.

The course of VIS in survivors and non-survivors is pairwise compared using non-parametric Wilcoxon method. Non-parametric comparison for all pairs is then performed using Dunn method for joint ranking with Bonferroni adjustment.

The relationship between pre-ECMO VIS and the selected ECMO modality (V-V and V-VA ECMO) was first analyzed with a logistic regression and receiver operating characteristic (ROC) curve analysis. The impact of V-VA ECMO in affecting VIS course and ICU mortality was also assessed.

Risk factors for mortality were evaluated using univariate analysis. The following factors were included in the outcome analysis: age, sex, body mass index, length of ICU stay, ECMO cannulation strategy, year and length of ECMO support, relevant comorbidities (septic shock, prior cardiac arrest and cardiac failure, preexisting chronic cardiopulmonary diseases), SAPS II, SOFA score and VIS.

Risk factors with *p* ˂ 0.05 in univariate analysis were then included in multi-variable analysis. The cut-off values of SAPS II, SOFA score and VIS for predicting mortality were determined through ROC curve analysis. Survival was estimated using Kaplan–Meier and Cox proportional hazard model.

Statistical analysis was performed with JMP^®^ Version 15 (SAS, Cary, NC, USA). A *p*-value of <0.05 was considered statistically significant.

## 3. Results

We included 221 ECMO runs (39 V-VA and 182 V-V ECMO) on 214 patients between November 2014 and July 2020 in the analysis. Five patients required V-V ECMO twice and one patient required three V-V ECMO runs due to recurring respiratory failure. We included 172 patients with primary ARDS because of bacterial or viral pneumonia, 38 patients with secondary ARDS due to sepsis (*n* = 8), systemic autoimmune disease (*n* = 6), peritonitis (*n* = 5), pancreatitis (*n* = 4), abdominal compartment (*n* = 4), trauma (*n* = 4), chemotherapy related (*n* = 2), heat stroke (*n* = 1), anaphylactic shock (*n* = 1), burn lesion (*n* = 1), postcardiotomy (*n* = 1) and chylothorax (*n* = 1), five patients with tracheal perforations and respiratory failure, five patients with pulmonary embolism and one patient with an aortopulmonary fistula.

### 3.1. Demographics and Characteristics

Patient demographics and characteristics in V-V and V-VA ECMO groups are outlined in Table 1. All V-V ECMOs were initiated due to respiratory failure, V-VA ECMOs were initiated in (1) primary respiratory failure with acute cor pulmonale or septic cardiomyopathy (85%) and (2) fulminant pulmonary embolism and aspiration pneumonia (15%).

Compared to patients with V-V ECMO, patients with V-VA ECMO had a higher pre-ECMO incidence of septic shock (85% vs. 56%, *p* = 0.0009), cardiac arrest (41% vs. 18%, *p* = 0.002) and cardiac failure (100% vs. 25%, *p* < 0.0001) and a higher SOFA (15 vs. 14, *p* = 0.002) and SAPS II score (86 vs. 76, *p* = 0.04). Patients with V-VA ECMO also demonstrated a shorter duration of ICU stay (13 vs. 21 days, *p* = 0.009) and a significantly higher ICU mortality (72% vs. 49%, *p* = 0.01). There was no statistical difference in age or sex between both ECMO groups (Table 1). ECMO was mostly initiated at ICU admission day for both V-V and V-VA ECMO group. Therefore, the median time interval between ICU admission and pre-ECMO data was less than 24 h. No patient underwent cardiac transplantation prior to or during ECMO support.

### 3.2. Vasoactive Inotropic Score (VIS)

The calculated VIS at ICU admission, pre-ECMO, during the first two days of ECMO and at the termination of ECMO support are presented in Table A1. Most patients received norepinephrine as vasopressor of choice, only two patients in V-VA ECMO group received vasopressin.

**Figure 1:**
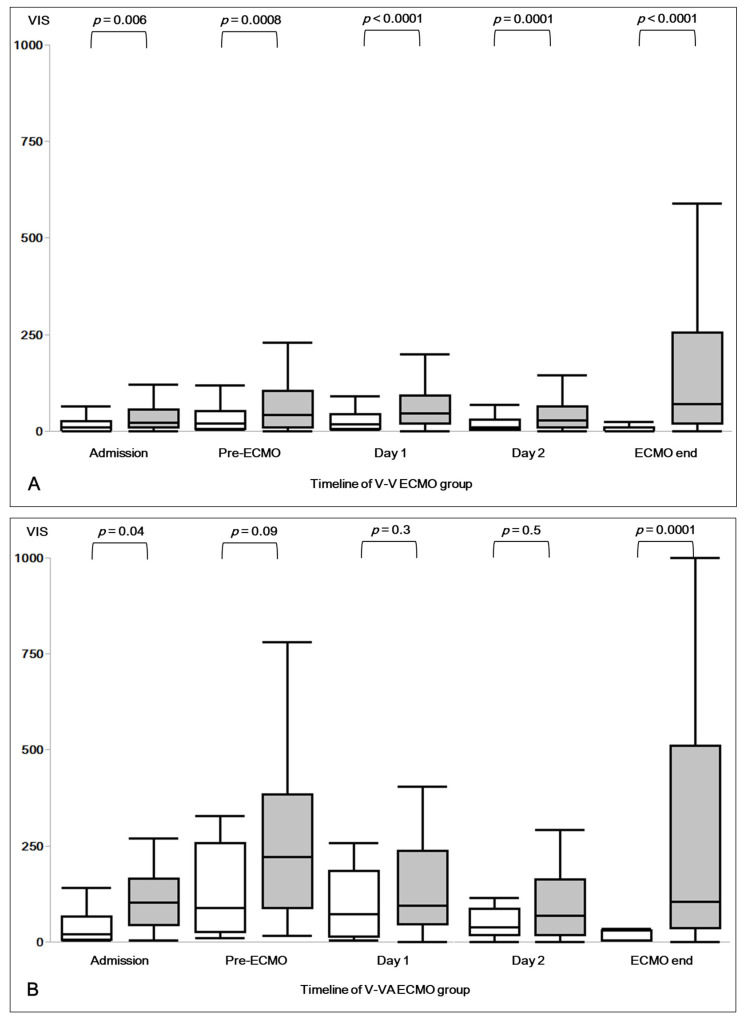
Trend in VIS in (**A**) V-V ECMO and (**B**) V-VA ECMO both for survivors (white) and non-survivors (gray). Boxplots show median and interquartile range Q1–Q3, brackets denote the differences between survivors and non-survivors, *p*-values are shown above the brackets. VIS = Vasoactive Inotropic Score; ECMO = Extracorporeal Membrane Oxygenation; V-V = Veno-Venous ECMO; V-VA = Veno-VenoArterial ECMO; pre-ECMO: immediately prior to ECMO initiation; ECMO end: immediately prior to the termination of ECMO support.

Pre-ECMO VIS was 40 (10–113) in all patients, 30 (10–80) in patients with V-V ECMO, and 207 (60–328) in patients with V-VA ECMO (Table A1). Patients under V-VA ECMO showed significantly higher VIS at every assessment time points (Table A1). Figure 1A,B present the VIS course in V-V and V-VA ECMO groups.

### 3.3. Predictive Performance of SAPS II, SOFA Score and VIS

In V-V and V-VA ECMO groups, we found a statistically significant difference in SAPS II in non-survivors (80 (72–91) vs. 91 (77–101), *p* = 0.026) but not in survivors (72 (64–82) vs. 67 (53–87), *p* = 0.30) (Table 2A).

Among all patients, we found an association between SAPS II and ICU mortality (*p* < 0.0001, ROC AUC 0.68) (Table 3). This association also occurred in logistic regression analyses of V-V ECMO (*p* = 0.0008, ROC AUC 0.65) and V-VA ECMO patients (*p* = 0.002, ROC AUC 0.80). We found a cut-off value of SAPS II for predicting mortality of 75 for all patients, 74 for patients with V-V ECMO and 76 for patients with V-VA ECMO (Table 2A). Multi-variable analysis failed to confirm SAPS II as an independent predictor of mortality (*p* = 0.5) (Table 3).

Non-survivors had a median SOFA score of 14 (12–17) and 16 (14–18) for V-V and V-VA ECMO (*p* = 0.001), respectively. Survivors did not show statistically significant differences between both ECMO modalities (*p* = 0.9) (Table 2B).

The cut-off values of SOFA score in predicting mortality were 13 among all patients (*p* = 0.0003, ROC AUC 0.64), 13 in V-V ECMO (*p* = 0.03, ROC AUC 0.59) and 15 in V-VA ECMO groups (*p* = 0.001, ROC AUC 0.79) (Table 2B).

Among all patients, SOFA score was associated with mortality in univariate analysis (*p* = 0.0003, ROC AUC 0.64) but not in multi-variable analysis (*p* = 0.1) (Table 3).

Non-survivors showed a median pre-ECMO VIS of 42 (11–105) and 222 (88–383) in V-V and V-VA ECMO groups, respectively (*p* < 0.0001; Table 2C). Survivors, on the other hand, showed a median pre-ECMO VIS of 19 (6–53) and 89 (27–257) in V-V and V-VA ECMO groups (*p* = 0.0008; Table 2C).

In univariate analysis, we found an association between pre-ECMO VIS and mortality (*p* < 0.0001, ROC AUC 0.68; Table 3). This association is particularly significant in the V-V ECMO group (*p* = 0.001, ROC AUC 0.64) but not in the V-VA ECMO group (*p* = 0.18, ROC AUC 0.68). The pre-ECMO VIS cut-off value for predicting mortality was 61.40 for all patients and patients with V-V ECMO, and 114.67 for patients with V-VA ECMO (Table 2C).

In multi-variable analysis, pre-ECMO VIS is an independent risk factor for mortality (*p* = 0.02; Table 3).

### 3.4. Survival Analysis

**Figure 2:**
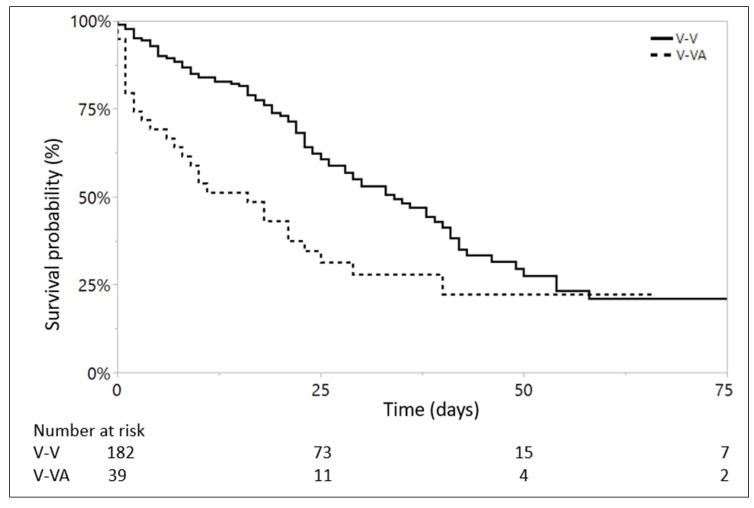
Kaplan–Meier curve for V-V and V-VA ECMO groups, Log-Rank *p* = 0.002. ECMO = Extracorporeal Membrane Oxygenation; V-V = Veno-Venous ECMO; V-VA = Veno-VenoArterial ECMO.

Kaplan–Meier estimates that the V-VA ECMO group has a shorter median survival time (16 days), as compared to V-V ECMO (34 days) (Log-Rank *p* = 0.002; Figure 2). Cox proportional hazard model also estimates that during ICU stay there are 1.92 more survivors in the V-V ECMO group than in the V-VA ECMO group (*p* = 0.003). However, both V-VA and V-V ECMO groups show similar 25% cumulative survival probability at two months after ECMO initiation (Figure 2).

### 3.5. Pre-ECMO VIS and ECMO Cannulation Strategy

In retrospective analysis, pre-ECMO VIS independently predicted our chosen cannulation strategy (*p* < 0.0001, ROC AUC 0.82). We preferred V-VA ECMO modality in patients with high VIS with a retrospectively calculated cut-off value of 100.5. Considering a cut-off value of 100.5 in selecting V-VA ECMO as support modality, the sensitivity was 67%, the specificity was 82%, the positive predictive value was 45% and the negative predictive value was 92%.

### 3.6. The Impact of V-VA ECMO in Patients with High Pre-ECMO VIS

Pre-ECMO VIS in patients with V-V ECMO was clearly associated with mortality (*p* = 0.001, ROC AUC 0.64). However, V-VA ECMO disrupted this association (*p* = 0.18, ROC AUC 0.68; Table 2).

V-VA survivors had a significantly higher pre-ECMO VIS compared to V-V ECMO non-survivors (Figure 1A,B). Furthermore, V-VA ECMO significantly reduced VIS during the first 48 h on ECMO (Figure 1B).

## 4. Discussion

The major findings of this study are (1) pre-ECMO VIS is an independent predictor of survival, (2) a high pre-ECMO VIS might indicate the requirement of V-VA ECMO, (3) V-VA ECMO reduces VIS during the first 48 h on ECMO support and (4) V-VA ECMO group has a shorter median estimated survival time but a similar survival probability as V-V ECMO group at two months after ECMO initiation.

To our knowledge, there is no validated scoring system for adults with (cardio-) respiratory failure to determine the optimal peripheral ECMO cannulation strategy. The type of ECMO support in primary respiratory failure should be selected based on hemodynamic stability and echocardiographic findings. Pre-ECMO VIS might be useful, as it (1) is easily determined at bedside with readily available parameters, (2) reflects the level of hemodynamic stability, (3) acceptably predicts mortality and (4) might discriminate suitable candidates for advanced cannulation strategies such as V-VA ECMO. Our study provides a first step in validating pre-ECMO VIS for these specific purposes.

### 4.1. Predictive Performance of SAPS II, SOFA Score and VIS

Previous studies reported that VIS predicts outcome in adult and pediatric cardiac surgery [17,18]. Nevertheless, the application of VIS in adult patients with severe cardiorespiratory dysfunction prior to and during ECMO support has not been evaluated.

Lee et al. reported a 27.32% survival rate in patients with a SAPS II score ˃70 before V-A ECMO initiation for cardiogenic shock [19]. Similarly, our patients on V-VA ECMO due to concomitant cardiopulmonary failure showed a median SAPS II score of 86 (68–97) and 28% survival rate. In contrast, patients on V-V ECMO had a lower SAPS II score of 76 (68–88) and a higher survival rate of 51%. Laimoud et al. reported that an initial SOFA score ≥13 had a 85% sensitivity and 73.9% specificity (*p* < 0.001, ROC AUC 0.86) for predicting hospital mortality in patients with cardiogenic shock supported with V-A ECMO [20]. Our patients with V-VA ECMO showed an initial median SOFA score of 15 and a mortality rate of 72%. Patients on V-V ECMO had a SOFA score of 14 and a mortality rate of 49%. These findings indicate a superior predictive performance of the SAPS II and SOFA scores in patients with V-A and V-VA ECMO support, as compared to V-V ECMO. As described by Fisser et al., this superiority might be due to the inclusion of more variables reflecting cardiac than respiratory parameters in SAPS II and SOFA scores [21]. This aspect might also cause the lack of statistical significance of both scores in our multi-variable analysis.

Vogel et al. reported a 25% mortality rate in 12 adults who supported by V-VA ECMO due to respiratory failure and septic cardiomyopathy [9]. Their patients had an initial SOFA score of 10, however, no SAPS II score and no further details on vasoactive-inotropic drug requirement was reported [9]. Based on the reported levosimendan and noradrenalin dosage, the calculated VIS in their study was 82.

Our study shows a 72% mortality rate in 39 patients with V-VA ECMO support, who had an initial SOFA score of 15 and a SAPS II score of 86. The last is associated with an estimated ICU mortality of 95.4%. The observed median pre-ECMO VIS in our V-VA ECMO group was 207. Retrospectively, V-VA ECMO was first initiated in patients with a pre-ECMO VIS ≥ 100.5. Both SOFA score and pre-ECMO VIS in our V-VA ECMO group were significantly higher than those reported by Vogel et al.

### 4.2. Pre-ECMO VIS and ECMO Cannulation Strategy

In our analysis, SAPS II, SOFA score and pre-ECMO VIS show an acceptable overall mortality prediction. However, the cut-off values of SAPS II on V-V ECMO and V-VA ECMO were 74 and 76, which predicted 88% and 89.7% mortality rate, respectively. The interval from each cut-off values and their predicted mortality rate is very small. Therefore, SAPS II might be less suitable in helping clinicians decide which ECMO modality makes the most sense to use. Cut-off values of SOFA score for predicting mortality were 13 and 15 for V-V ECMO and V-VA ECMO group. The interval from each cut-off value is also very small and, thus, not particularly helpful in selecting ECMO modality.

Furthermore, laboratory results might not always be available to calculate SAPS II or SOFA score in a high-urgency setting. Here, VIS has practical advantage. Pre-ECMO VIS is easily calculated at the bedside, assesses the required hemodynamic support [11] and offers assistance in selecting the optimal ECMO modality.

Our analyses suggest that pre-ECMO VIS can help to select the optimal ECMO modality. Retrospectively, we analyzed our institutional preference towards V-VA ECMO in patients with pre-ECMO VIS ≥ 100.5 (*p* < 0.0001, ROC AUC 0.82). It had acceptable sensitivity and specificity of 67% and 82%. The low positive predictive value of 45% and the high negative predictive value of 92% were likely related to the small number of V-VA ECMO cases.

V-VA ECMO has been shown to support hemodynamics in patients with cardiopulmonary failure [6,9]. This observation is consistent with our results. After ECMO initiation, there is a tendency for VIS to decrease in survivors, but not in non-survivors. Patients who do not wean from vasopressor and inotropic support rapidly after ECMO initiation are patients with severe or persistent hemodynamic instability and are more likely to have a poor outcome. Figure 1B also illustrates the ability of V-VA ECMO to reduce the required vasoactive-inotropic drugs on ECMO survivors. V-V ECMO on the other hand (Figure 1A), does not seem to reduce VIS as effective as V-VA ECMO, both on survivors and non-survivors. Based on pre-ECMO VIS, survival of V-V ECMO non-survivors might be improved through V-VA ECMO.

### 4.3. The Impact of V-VA ECMO on Patients with High Pre-ECMO VIS

In our study, a pre-ECMO VIS of greater than 61.4 identifies patients with significantly lower odds of survival. Among V-VA ECMO patients, the pre-ECMO VIS cut-off value of 114.67, and the disrupted relationship between pre-ECMO VIS and mortality might reflect the ability of V-VA ECMO to reduce the requirement of vasoactive-inotropic drugs in a greater extent than V-V ECMO. In line with our findings, the European Society of Pediatric and Neonatal Intensive Care recognizes that VIS greater than 200 is associated with mortality and the requirement of V-A ECMO support in children with refractory septic shock [13]. Brechot et al. reported a reduction in inotropic score, which is solely based on dobutamine, epinephrine and norepinephrine from 250 to 14 within the first 12 h of V-A ECMO support in adult refractory cardiovascular dysfunction during severe bacterial septic shock [22]. Our data only show a VIS reduction from 207 to 77 within the first 24 h of V-VA ECMO. This might be explained by the use of levosimendan which forms an active metabolite that sustains its inodilator effects for up to a week [23]. In this study, we integrated the dose of levosimendan to the VIS calculation for a week.

In Kaplan–Meier analysis, the V-VA ECMO group, which has a significantly higher pre-ECMO VIS, has a shorter median survival time as compared to V-V ECMO group (16 vs. 34 days). However, both V-VA and V-V ECMO groups show similar 25% cumulative survival probability at two months after ECMO initiation. This last finding might reflect the ability of V-VA ECMO to support highly unstable patients with cardiorespiratory failure.

Furthermore, in agreement with Vogel et al., our findings suggest that earlier implementation of V-VA ECMO on patients with lower pre-ECMO VIS might improve survival. As illustrated in Figure 1A,B, V-V ECMO non-survivors had a significantly lower pre-ECMO VIS as compared to V-VA survivors and, thus, might benefit from early V-VA support.

We propose a routine VIS assessment to quantify hemodynamic alterations prior to and during ECMO support. A transition from V-V to V-VA ECMO might be evaluated if a VIS of 61 is reached. However, a pre-ECMO VIS of greater than 115 substantially reduces the odds of survival, irrespective of the cannulation strategy.

Notably, the pre-ECMO VIS of V-VA ECMO survivors was significantly higher than VIS of V-V ECMO non-survivors (89 vs. 42). Here, V-VA ECMO seems to disrupt the association between pre-ECMO VIS and mortality due to factors mentioned previously.

### 4.4. Limitations

Our analysis shares the limitations of a retrospective review and its selection bias. Due to the small number of cases, particularly in the V-VA ECMO group, individual cases are only partially comparable, and the study population may not be large enough to pick up a statistically significant difference.

Furthermore, we acknowledge the fact that VIS as calculated in this study reflects (1) our institutional ECMO management, (2) our preferred method in the optimization of cardiac pre- and afterload and (3) our preferred inotropic agent, as outlined in Appendix A. Therefore, our results might not be applicable for other centers.

Of note, our workflow lacked a validated preload determinant during V-VA ECMO support. In our ECMO center, preload status and fluid therapy were determined through the passive leg raised test in conjunction with echocardiographic assessment. Passive leg raised test was chosen as a substitute for volume expansion which has been validated in one single center study in patients managed with V-V ECMO [24] but not in patients supported with V-VA ECMO.

Lastly, we evaluated a cohort with primary respiratory failure (i.e., severe ARDS) and concomitant, secondary cardio-circulatory failure due to septic shock or acute cor pulmonale. In ARDS, septic shock and acute cor pulmonale, the right ventricle is the main limiting factor of cardiac fluid-unresponsiveness and circulatory failure [25,26]. Thus, our methods and findings might not be applied to patients with primary left heart failure leading to cardiogenic shock.

## 5. Conclusions

The optimal ECMO cannulation strategy in primary respiratory failure should be selected based on hemodynamic stability and echocardiographic findings. VIS is easily calculated, reflects hemodynamic stability and independently predicts mortality in patients managed with ECMO. In conjunction with echocardiography, VIS might assist clinicians in caring for patients with severe (cardio-) pulmonary failure. In patients with VIS higher than 61 immediately prior to ECMO initiation, a Veno-VenoArterial cannulation strategy might be reasonable. Despite the significant hemodynamic compromise in the V-VA group, both cannulation modalities show a similar survival probability at two months after ECMO initiation. This finding might reflect the ability of V-VA ECMO to support highly unstable patients with combined cardiorespiratory failure.

## Figures and Tables

**Table 1:** Patient demographics and characteristics in V-V and V-VA ECMO groups.

	V-V ECMO (*n* = 182)	V-VA ECMO (*n* = 39)	
Age (years)	56 (46–63)	60 (49–63)	*p* = 0.62
Male sex (%)	*n* = 121 (66%)	*n* = 29 (74%)	*p* = 0.45
Body weight (kg)	85 (75–105)	80 (70–90)	***p* = 0.035**
Clinical presentation prior to ECMO initiation respiratory failure septic shock cardiac comorbidities cardiac failure cardiac arrest	*n* = 182 (100%) *n* = 102 (56%) *n* = 74 (41%) *n* = 46 (25%) *n* = 32 (18%)	*n* = 33 (85%) *n* = 33 (85%) *n* = 39 (100%) *n* = 39 (100%) *n* = 16 (41%)	***p* < 0.0001** ***p* = 0.0009** ***p* < 0.0001** ***p* < 0.0001** ***p* = 0.002**
Time from ICU admission to ECMO initiation (days)	0 (0–1)	0 (0–1)	*p* = 0.7
Duration of ECMO support (days)	13 (8–21)	7 (1–15)	***p* < 0.0001**
Duration of arterial support (days)		5 (1–7)	
ICU length of stay (days)	21 (13–33)	13 (2–25)	***p* = 0.009**
SAPS II score within 24 h after ICU admission	76 (68–88)	86 (68–97)	***p* = 0.04**
Predicted mortality based on median SAPS II score	89.7%	95.4%	
SOFA score within 24 h after ICU admission	14 (12–16)	15 (13.5–18)	***p* = 0.002**
Predicted mortality based on median SOFA score	60%	˃80%	
ICU mortality (%)	*n* = 89 (49%)	*n* = 28 (72%)	***p* = 0.01**
Mortality cause: Cardiovascular failure Non-cardiovascular organ failure ECMO related failure	*n* = 30 (16%) *n* = 54 (30%) *n* = 5 (3%)	*n* = 14 (36%) *n* = 11 (28%) *n* = 3 (8%)	***p* = 0.01** *p* = 1.0 *p* = 0.1

Data is presented as median (interquartile range Q1–Q3) or *n* (%). Bold *p*-values express statistically significant differences between V-V and V-VA ECMO groups. ECMO = Extracorporeal Membrane Oxygenation; V-V = Veno-Venous; V-VA = Veno-VenoArterial; SAPS II = Simplified Acute Physiology Score II; SOFA = Sequential Organ Failure Assessment; ICU: intensive care unit.

**Table 2:** (**A**) SAPS II within the first 24 h after ICU admission in all patients, non-survivors and survivors. (**B**) SOFA score within the first 24 h after ICU admission in all patients, non-survivors and survivors. (**C**) Pre-ECMO VIS in all patients, non-survivors and survivors.

(**A**)				
**SAPS II**	**All Patients**	**V-V ECMO**	**V-VA ECMO**	
Non-survivors	82 (72–93)	80 (72–91)	91 (77–101)	***p* = 0.026**
Survivors	72 (63–82)	72 (64–82)	67 (53–87)	*p* = 0.30
Cut-off value	75	74	76	
	***p* < 0.0001**	***p* = 0.0008**	***p* = 0.002**	
(**B**)				
**SOFA Score**	**All Patients**	**V-V ECMO**	**V-VA ECMO**	
Non-survivors	14 (13–17)	14 (12–17)	16 (14–18)	***p* = 0.001**
Survivors	13 (11–15)	13 (11–15)	13.5 (10–15)	*p* = 0.9
Cut-off value	13	13	15	
	***p* = 0.0004**	***p* = 0.03**	***p* = 0.001**	
(**C**)				
**VIS**	**All Patients**	**V-V ECMO**	**V-VA ECMO**	
Non-survivors	61 (18–174)	42 (11–105)	222 (88–383)	***p* < 0.0001**
Survivors	20 (8–60)	19 (6–53)	89 (27–257)	***p* = 0.0008**
Cut-off value	61.40	61.40	114.67	
	***p* < 0.0001**	***p* = 0.001**	*p* = 0.18	

All scores are shown in median (interquartile range Q1–Q3); *p*-values on the right denotes differences between V-V and V-VA ECMO in survivors and non-survivors. *p*-values below the cut-off values denote differences between survivors and non-survivors within each ECMO group. Bold *p*-values represent statistically significant differences. ECMO = Extracorporeal Membrane Oxygenation; V-V = Veno-Venous ECMO; V-VA = Veno-VenoArterial ECMO; SAPS II = Simplified Acute Physiology Score II; SOFA = Sequential Organ Failure Assessment; VIS = Vasoactive Inotropic Score; ICU = Intensive Care Unit.

**Table 3:** Factors affecting overall ICU mortality in univariate and multi-variable analyses.

	Univariate Analysis	Multi Variable Analysis
**ECMO-Type (V-V and V-VA ECMO)**	***p* = 0.01**	*p* = 0.9
**Presence of septic shock**	***p* = 0.02**	*p* = 0.9
**Presence of cardiac morbidities**	***p* = 0.0002**	*p* = 0.2
**Presence of cardiac failure** **(acute cor pulmonale, septic cardiomyopathy)**	***p* < 0.0001**	*p* = 0.05
**Presence of cardiac arrest**	***p* < 0.0001**	***p* = 0.0002**
**Pre-ECMO VIS**	***p*< 0.0001**; ROC AUC 0.68	***p* = 0.02**
**SOFA score**	***p*= 0.0003**; ROC AUC 0.64	*p* = 0.1
**SAPS II**	***p*< 0.0001**; ROC AUC 0.68	*p* = 0.5
**ICU length of stay**	***p*= 0.0002**; ROC AUC 0.66	***p* < 0.0001**
**Length of ECMO support**	*p* = 0.55	
**Year of ECMO support**	***p* = 0.01**	*p* = 0.5
**Age (year)**	***p* = 0.004**; ROC AUC 0.61	*p* = 0.1

Bold numbers represent statistical significance; SAPS II = Simplified Acute Physiology Score II; SOFA = Sequential Organ Failure Assessment; VIS = Vasoactive Inotropic Score; ECMO = Extracorporeal Membrane Oxygenation; V-V = Veno-Venous ECMO; V-VA = Veno-VenoArterial ECMO; pre-ECMO: immediately prior to ECMO initiation; ICU = Intensive Care Unit; ROC AUC Receiver Operating Characteristic Area Under (the ROC) Curve.

## Data Availability

The analyzed datasets for this study are available from the corresponding author upon reasonable request.

## Appendix A. Institutional Management Strategy for Patients on ECMO Support

### Appendix A.1. Indications for V-V ECMO

In agreement with the guidelines established by the Extracorporeal Life Support Organization (ELSO) [27] and the ECMO to rescue Lung Injury in severe ARDS (EOLIA) trial [2], ECMO is initiated in fully sedated patients (Richmond Agitation-Sedation Scale −5) with

- arterial partial pressure of oxygen / fraction of inspired oxygen (PaO_2_/FiO_2_) less than 50 mmHg for longer than 3 h, or
- PaO_2_/FiO_2_ less than 80 mmHg for longer than 6 h or
- persistent acidosis (arterial pH less than 7.25 and arterial partial pressure of carbon dioxide (PaCO_2_) greater than 60 mmHg for longer than 6 h),
  despite protective mechanical ventilation (a tidal volume of 6 mL/kg, a positive end expiratory pressure (PEEP) adjustment according to the lowest elastance of the respiratory system and a driving pressure less than 15 cm H_2_O) and prone positioning [2,28,29,30,31]. Prone positioning and neuromuscular blocking agents are prescribed according to the attending physician.

Furthermore, ECMO is considered in severe ARDS persistence without clinical improvement, despite protective mechanical ventilation and at least two cycles of prone positioning [2,32].

### Appendix A.2. Indications for V-VA ECMO

V-VA ECMO is initiated in cases with concomitant cardiopulmonary failure (i.e., meeting the V-V ECMO indications as described previously AND persistent tissue hypoperfusion, systolic blood pressure less than 90 mmHg, cardiac index less than 2.0 L/min/m^2^, while receiving norepinephrine greater than 0.5 µg/kg/min, dobutamine greater than 20 µg/kg/min or equivalent and adequate cardiac preload).

Tissue hypoperfusion is identified with an increased arterial lactate level greater than 2 mmol/L. Cardiac preload status (i.e., fluid responsiveness) is determined by a passive leg raised test and stroke volume measurement with echocardiography [24]. Passive leg raising induced changes in stroke volume greater than 10% indicates fluid responsiveness [24].

As the right ventricle is the main limiting factor of cardiac fluid-unresponsiveness and circulatory failure in ARDS, septic shock and acute cor pulmonale [25,26], further echocardiographic assessment is completed to rule out right or left ventricular failure.

Right ventricular failure is clinically identified by a combination of systemic hypoperfusion and systemic congestion with central venous pressure (CVP) greater than 8 mmHg [10]. Echocardiographic signs are

- an increased systolic pulmonary artery pressure (sPAP), which is calculated at end-expiration based on the maximal velocity (Vmax) of the tricuspid regurgitation (sPAP = 4 × Vmax2 + CVP),
- a dilated right ventricle (ratio of the RV/LV end-diastolic area ≥ 0.6) in four chamber view and
- the presence of an interventricular septal dyskinesia in the short-axis view of the heart [25,33].

The presence of interventricular septal dyskinesia is termed cor pulmonale [26].

Left ventricular ejection fraction (LVEF) and cardiac output are assessed with Simpson’s biplane method and velocity time integral at the left ventricular outflow tract. A reduced LVEF of less than 40% and a calculated cardiac index less than 2.0 L/min/m^2^ are considered as a failing left ventricle [34,35].

In patients with cardiac failure, levosimendan is infused at a rate of 0.1 µg/kg/min. Vasopressor (norepinephrine) and other inotropic drugs (dobutamine or epinephrine) are adjusted to achieve an arterial lactate level less than 2 mmol/L, a mean arterial pressure of 65 mmHg and a cardiac index greater than 2 L/min/m^2^, which is routinely evaluated with echocardiography. The chosen inotropic agent depends on patient’s underlying disease and the clinician’s preference. In cases with catecholamine refractory shock, V-VA ECMO is initiated. We prefer to support these patients with V-VA ECMO to avoid upper body hypoxemia.

To optimize the cardiac afterload and guide vasopressor therapy, the systemic vascular resistance index is calculated as follows [36]

$$\mathrm{SVRI}\;\left({\mathrm{dyn}*s*\mathrm{cm}}^{-5}*m^{2}\right)=\left[\frac{\left(\mathrm{mean}\;\mathrm{arterial}\;\mathrm{pressure}-\mathrm{central}\;\mathrm{venous}\;\mathrm{pressure}\right)}{\mathrm{cardiac}\;\mathrm{index}}\right]\times 80$$

SVRI values between 1800 and 2400 are considered normal.

### Appendix A.3. Cannulation Strategy

The standard cannulation uses a 29 Fr multi-stage drainage cannula and a 23 Fr venous return cannula, which are inserted through the right femoral and jugular veins, respectively. V-VA ECMO is initiated with an additional 17 Fr arterial cannula and a 7 Fr leg perfusion cannula, which are inserted through the left femoral artery.

### Appendix A.4. ECMO Management

The ECMO blood and gas flow are adjusted to obtain an arterial partial pressure of oxygen (PaO_2_) between 65 and 90 mmHg, an arterial partial pressure of carbon dioxide (PaCO_2_) under 45 mmHg and an arterial pH of 7.35–7.45 [2].

During ECMO support, the ventilator is set to a volume-controlled mode with a tidal volume of 2 mL per kilogram of ideal bodyweight, a respiratory rate of 12 per minute and a fraction of inspired oxygen of 40%. PEEP is titrated according to the lowest elastance of the respiratory system, as described in our previous report [31].

If arterial lactate level starts to increase (greater than 2 mmol/L) under ECMO support, a passive leg raised test in conjunction with echocardiographic assessment is performed to evaluate cardiac preload and fluid responsiveness. A lack of fluid responsiveness requires further echocardiographic assessment to rule out right or left ventricular failure.

In patients managed with V-VA ECMO, daily echocardiography is performed to ensure aortic valve opening and to rule out left ventricular distension and mitral regurgitation. If we detect cardiac recovery, the flow rate on the arterial cannula is gradually reduced to a minimum of 1 L/min. After 24 h of hemodynamic stability with an arterial flow of 1 L/min, the arterial canula is surgically removed.

A positive fluid balance in patients with ARDS has been associated with an increased mortality and duration of mechanical ventilation [37,38,39]. Thus, after an initial ECMO stabilization period, we use diuretics or hemodialysis to facilitate a negative fluid balance. Afterward, we promote spontaneous breathing by tapering the analgosedation. If clinically feasible, an ECMO weaning trial is performed by reducing the ECMO gas flow to 0 L/min for at least 24 h. ECMO support is discontinued if the PaO_2_ is higher than 70 mmHg and the arterial pH is greater than 7.25, with fraction of inspired oxygen less than 60% and an inspiratory plateau pressure less than 30 cm H_2_O having been achieved [2].

**Table A1:** VIS assessment at various time points.

VIS Assessment Timeline	All Patients	V-V ECMO	V-VA ECMO	
ICU admission	18 (5–60)	13 (4–40)	73 (20–134)	***p* = 0.002**
Pre-ECMO	40 (10–113)	30 (10–80)	207 (60–328)	***p* < 0.0001**
Day 1	32 (10–80)	29 (10–69)	77 (41–215)	***p* < 0.0001**
Day 2	20 (8–52)	20 (7–50)	53 (18–97)	***p* = 0.03**
ECMO end	20 (0–97)	10 (0–71)	50 (10–422)	***p* = 0.03**

All values are presented as median (interquartile range Q1–Q3). Bold numbers represent statistically significant differences. VIS = Vasoactive Inotropic Score; ECMO: Extracorporeal Membrane Oxygenation; V-V: Veno-Venous; V-VA: Veno-VenoArterial; ICU: intensive care unit; pre-ECMO: immediately prior to ECMO initiation; ECMO end: immediately prior to the termination of ECMO support.

**Table A2:** Causality assessment of using vasoactive inotropic drugs prior to ECMO initiation.

Vasoactive Inotropic Drugs	V-V ECMO (*n* = 182)	V-VA ECMO (*n* = 39)
**Dopamine**	*n* = 0	*n* = 0
**Dobutamine**	*n* = 15 decreased LV function *n* = 5 decreased RV function *n* = 10	*n* = 10 decreased LV function *n* = 7 decreased RV function *n* = 2 decreased biventricular function *n* = 1
**Epinephrine**	*n* = 5 decreased LV function *n* = 3 decreased RV function *n* = 2	*n* = 9 decreased LV function *n* = 5 decreased RV function *n* = 1 decreased biventricular function *n* = 3
**Norepinephrine**	*n* = 160 MAP < 65 mmHg	*n* = 39 MAP < 65 mmHg
**Milrinone**	*n* = 0	*n* = 0
**Vasopressin**	*n* = 0	*n* = 2 MAP < 65 mmHg
**Levosimendan**	*n* = 7 decreased LV function *n* = 4 decreased RV function *n* = 3	*n* = 20 decreased LV function *n* = 11 decreased RV function *n* = 5 decreased biventricular function *n* = 4

All values are presented as *n* (%). ECMO: Extracorporeal Membrane Oxygenation; V-V: Veno-Venous; V-VA: Veno-VenoArterial; LV: left ventricular; RV: right ventricular; MAP: mean arterial pressure.

## References

[1] Force A.D.T., Ranieri V.M., Rubenfeld G.D., Thompson B.T., Ferguson N.D., Caldwell E., Fan E., Camporota L., Slutsky A.S. (2012). Acute respiratory distress syndrome: The Berlin Definition. JAMA.

[2] Combes A., Hajage D., Capellier G., Demoule A., Lavoue S., Guervilly C., Da Silva D., Zafrani L., Tirot P., Veber B. (2018). Extracorporeal Membrane Oxygenation for Severe Acute Respiratory Distress Syndrome. N. Engl. J. Med..

[3] Goligher E.C., Tomlinson G., Hajage D., Wijeysundera D.N., Fan E., Jüni P., Brodie D., Slutsky A.S., Combes A. (2018). Extracorporeal membrane oxygenation for severe acute respiratory distress syndrome and posterior probability of mortality benefit in a post hoc bayesian analysis of a randomized clinical trial. JAMA.

[4] Combes A., Peek G.J., Hajage D., Hardy P., Abrams D., Schmidt M., Dechartres A., Elbourne D. (2020). Ecmo for severe ards: Systematic review and individual patient data meta-analysis. Intensive Care Med..

[5] Kon Z.N., Bittle G.J., Pasrija C., Pham S.M., Mazzeffi M.A., Herr D.L., Sanchez P.G., Griffith B.P. (2017). Venovenous versus Venoarterial Extracorporeal Membrane Oxygenation for Adult Patients with Acute Respiratory Distress Syndrome Requiring Precannulation Hemodynamic Support: A Review of the ELSO Registry. Ann. Thorac. Surg.

[6] Ius F., Sommer W., Tudorache I., Avsar M., Siemeni T., Salman J., Puntigam J., Optenhoefel J., Greer M., Welte T. (2015). Veno-veno-arterial extracorporeal membrane oxygenation for respiratory failure with severe haemodynamic impairment: Technique and early outcomes. Interact. Cardiovasc. Thorac. Surg..

[7] Napp L.C., Kuhn C., Hoeper M.M., Vogel-Claussen J., Haverich A., Schafer A., Bauersachs J. (2016). Cannulation strategies for percutaneous extracorporeal membrane oxygenation in adults. Clin. Res. Cardiol..

[8] Lusebrink E., Orban M., Kupka D., Scherer C., Hagl C., Zimmer S., Luedike P., Thiele H., Westermann D., Massberg S. (2020). Prevention and treatment of pulmonary congestion in patients undergoing venoarterial extracorporeal membrane oxygenation for cardiogenic shock. Eur. Heart J..

[9] Vogel D.J., Murray J., Czapran A.Z., Camporota L., Ioannou N., Meadows C.I.S., Sherren P.B., Daly K., Gooby N., Barrett N. (2018). Veno-arterio-venous ECMO for septic cardiomyopathy: A single-centre experience. Perfusion.

[10] Gaies M.G., Jeffries H.E., Niebler R.A., Pasquali S.K., Donohue J.E., Yu S., Gall C., Rice T.B., Thiagarajan R.R. (2014). Vasoactive-inotropic score is associated with outcome after infant cardiac surgery: An analysis from the Pediatric Cardiac Critical Care Consortium and Virtual PICU System Registries. Pediatr. Crit. Care Med..

[11] Favia I., Vitale V., Ricci Z. (2013). The vasoactive-inotropic score and levosimendan: Time for LVIS?. J. Cardiothorac. Vasc. Anesth..

[12] Koponen T., Karttunen J., Musialowicz T., Pietilainen L., Uusaro A., Lahtinen P. (2019). Vasoactive-inotropic score and the prediction of morbidity and mortality after cardiac surgery. Br. J. Anaesth..

[13] Morin L., Ray S., Wilson C., Remy S., Benissa M.R., Jansen N.J.G., Javouhey E., Peters M.J., Kneyber M., De Luca D. (2016). Refractory septic shock in children: A European Society of Paediatric and Neonatal Intensive Care definition. Intensive Care Med..

[14] Weiss S.L., Peters M.J., Alhazzani W., Agus M.S.D., Flori H.R., Inwald D.P., Nadel S., Schlapbach L.J., Tasker R.C., Argent A.C. (2020). Surviving sepsis campaign international guidelines for the management of septic shock and sepsis-associated organ dysfunction in children. Intensive Care Med..

[15] Le Gall J.R., Lemeshow S., Saulnier F. (1993). A new Simplified Acute Physiology Score (SAPS II) based on a European/North American multicenter study. JAMA.

[16] Vincent J.L., Moreno R., Takala J., Willatts S., De Mendonca A., Bruining H., Reinhart C.K., Suter P.M., Thijs L.G., On behalf of the Working Group on Sepsis-Related Problems of the European Society of Intensive Care Medicine (1996). The SOFA (Sepsis-related Organ Failure Assessment) score to describe organ dysfunction/failure. Intensive Care Med..

[17] Gaies M.G., Gurney J.G., Yen A.H., Napoli M.L., Gajarski R.J., Ohye R.G., Charpie J.R., Hirsch J.C. (2010). Vasoactive-inotropic score as a predictor of morbidity and mortality in infants after cardiopulmonary bypass. Pediatr. Crit. Care Med..

[18] Yamazaki Y., Oba K., Matsui Y., Morimoto Y. (2018). Vasoactive-inotropic score as a predictor of morbidity and mortality in adults after cardiac surgery with cardiopulmonary bypass. J. Anesth..

[19] Lee H.S., Kim H.S., Lee S.H., Lee S.A., Hwang J.J., Park J.B., Kim Y.H., Moon H.J., Lee W.S. (2019). Clinical implications of the initial SAPS II in veno-arterial extracorporeal oxygenation. J. Thorac. Dis..

[20] Laimoud M., Alanazi M. (2020). The Validity of SOFA Score to Predict Mortality in Adult Patients with Cardiogenic Shock on Venoarterial Extracorporeal Membrane Oxygenation. Crit. Care Res. Pract..

[21] Fisser C., Rincon-Gutierrez L.A., Enger T.B., Taccone F.S., Broman L.M., Belliato M., Nobile L., Pappalardo F., Malfertheiner M.V. (2021). Validation of Prognostic Scores in Extracorporeal Life Support: A Multi-Centric Retrospective Study. Membranes.

[22] Brechot N., Luyt C.E., Schmidt M., Leprince P., Trouillet J.L., Leger P., Pavie A., Chastre J., Combes A. (2013). Venoarterial extracorporeal membrane oxygenation support for refractory cardiovascular dysfunction during severe bacterial septic shock. Crit. Care Med..

[23] Lilleberg J., Laine M., Palkama T., Kivikko M., Pohjanjousi P., Kupari M. (2007). Duration of the haemodynamic action of a 24-h infusion of levosimendan in patients with congestive heart failure. Eur. J. Heart Fail..

[24] Guinot P.G., Zogheib E., Detave M., Moubarak M., Hubert V., Badoux L., Bernard E., Besserve P., Caus T., Dupont H. (2011). Passive leg raising can predict fluid responsiveness in patients placed on venovenous extracorporeal membrane oxygenation. Crit. Care.

[25] Vieillard-Baron A., Prigent A., Repesse X., Goudelin M., Prat G., Evrard B., Charron C., Vignon P., Geri G. (2020). Right ventricular failure in septic shock: Characterization, incidence and impact on fluid responsiveness. Crit. Care.

[26] Vieillard-Baron A., Naeije R., Haddad F., Bogaard H.J., Bull T.M., Fletcher N., Lahm T., Magder S., Orde S., Schmidt G. (2018). Diagnostic workup, etiologies and management of acute right ventricle failure: A state-of-the-art paper. Intensive Care Med..

[27] Tonna J.E., Abrams D., Brodie D., Greenwood J.C., Rubio Mateo-Sidron J.A., Usman A., Fan E. (2021). Management of Adult Patients Supported with Venovenous Extracorporeal Membrane Oxygenation (VV ECMO): Guideline from the Extracorporeal Life Support Organization (ELSO). ASAIO J..

[28] Amato M.B., Meade M.O., Slutsky A.S., Brochard L., Costa E.L., Schoenfeld D.A., Stewart T.E., Briel M., Talmor D., Mercat A. (2015). Driving pressure and survival in the acute respiratory distress syndrome. N. Engl. J. Med..

[29] Shekar K., Badulak J., Peek G., Boeken U., Dalton H.J., Arora L., Zakhary B., Ramanathan K., Starr J., Akkanti B. (2020). Extracorporeal Life Support Organization Coronavirus Disease 2019 Interim Guidelines: A Consensus Document from an International Group of Interdisciplinary Extracorporeal Membrane Oxygenation Providers. ASAIO J..

[30] Brower R.G., Lanken P.N., MacIntyre N., Matthay M.A., Morris A., Ancukiewicz M., Schoenfeld D., Thompson B.T., National Heart L., Blood Institute A.C.T.N. (2004). Higher versus lower positive end-expiratory pressures in patients with the acute respiratory distress syndrome. N. Engl. J. Med..

[31] Krebs J., Pelosi P., Rocco P.R.M., Hagmann M., Luecke T. (2018). Positive end-expiratory pressure titrated according to respiratory system mechanics or to ARDSNetwork table did not guarantee positive end-expiratory transpulmonary pressure in acute respiratory distress syndrome. J. Crit. Care.

[32] Schmidt M., Pham T., Arcadipane A., Agerstrand C., Ohshimo S., Pellegrino V., Vuylsteke A., Guervilly C., McGuinness S., Pierard S. (2019). Mechanical Ventilation Management during Extracorporeal Membrane Oxygenation for Acute Respiratory Distress Syndrome. An International Multicenter Prospective Cohort. Am. J. Respir. Crit. Care Med..

[33] Mekontso Dessap A., Boissier F., Charron C., Begot E., Repesse X., Legras A., Brun-Buisson C., Vignon P., Vieillard-Baron A. (2016). Acute cor pulmonale during protective ventilation for acute respiratory distress syndrome: Prevalence, predictors, and clinical impact. Intensive Care Med..

[34] Lang R.M., Badano L.P., Mor-Avi V., Afilalo J., Armstrong A., Ernande L., Flachskampf F.A., Foster E., Goldstein S.A., Kuznetsova T. (2015). Recommendations for cardiac chamber quantification by echocardiography in adults: An update from the American Society of Echocardiography and the European Association of Cardiovascular Imaging. Eur. Heart J.-Cardiovasc. Imaging.

[35] Blanco P. (2020). Rationale for using the velocity-time integral and the minute distance for assessing the stroke volume and cardiac output in point-of-care settings. Ultrasound J..

[36] Schoettler J.J., Kirschning T., Hagmann M., Hahn B., Fairley A.M., Centner F.S., Schneider-Lindner V., Herrle F., Tzatzarakis E., Thiel M. (2021). Maintaining oxygen delivery is crucial to prevent intestinal ischemia in critical ill patients. PLoS ONE.

[37] Shah A., Menaker J., Mazzeffi M.A., Galvagno S.M., Deatrick K.B., Madathil R.J., Rector R., O’Connor J.V., Scalea T.M., Tabatabai A. (2021). Association of Volume Status during Veno-Venous Extracorporeal Membrane Oxygenation with Outcome. ASAIO J..

[38] Wiedemann H.P., Wheeler A.P., Bernard G.R., Thompson B.T., Hayden D., de Boisblanc B., Connors A.F., Hite R.D., Harabin A.L., National Heart, Lung, and Blood Institute Acute Respiratory Distress Syndrome (ARDS) Clinical Trials Network (2006). Comparison of two fluid-management strategies in acute lung injury. N. Engl. J. Med..

[39] Seitz K.P., Caldwell E.S., Hough C.L. (2020). Fluid management in ARDS: An evaluation of current practice and the association between early diuretic use and hospital mortality. J. Intensive Care.

